# Oncologic Outcomes After ABO-Incompatible Versus Compatible Living Donor Liver Transplantation for Hepatocellular Carcinoma: A Systematic Review and Meta-Analysis

**DOI:** 10.3390/cancers18111687

**Published:** 2026-05-22

**Authors:** Seoung Hoon Kim, Byeong Ho An, Jin A Lee, Go Woon Jeong

**Affiliations:** Organ Transplantation Center, National Cancer Center, 111 Jungbalsan-ro, Ilsandong-gu, Goyang-si 10408, Republic of Korea; 70938@ncc.re.kr (B.H.A.); jina71@ncc.re.kr (J.A.L.); 11821@ncc.re.kr (G.W.J.)

**Keywords:** ABO-incompatible, living-donor liver transplantation, hepatocellular carcinoma, meta-analysis, recurrence-free survival, overall survival

## Abstract

**Background:** ABO-incompatible living-donor liver transplantation (ABOi LDLT) can expand the donor pool for patients with hepatocellular carcinoma (HCC), but concerns remain regarding tumor recurrence and long-term survival. **Methods:** A systematic review and meta-analysis was performed according to PRISMA 2020. PubMed, Embase, and Web of Science were searched. Comparative studies evaluating oncologic outcomes after ABOi versus ABO-compatible (ABOc) LDLT for HCC were included in the quantitative synthesis; non-comparative studies were included in the qualitative synthesis. Hazard ratios (HRs) for recurrence-free survival (RFS) and overall survival (OS) were pooled using a random-effects model. When HRs were not directly reported, they were estimated from Kaplan–Meier curves using established methods. **Results:** Sixteen reports were screened, 12 full-text articles were assessed, and 8 studies were included in the systematic review. Three comparative single-center cohort studies were eligible for meta-analysis. Pooled analysis showed no significant difference between ABOi and ABOc LDLT for RFS (HR 1.07, 95% confidence interval [CI] 0.77–1.49; I^2^ = 0%) or OS (HR 1.08, 95% CI 0.74–1.57; I^2^ = 0%). Five additional studies were synthesized qualitatively, suggesting that recurrence risk may be influenced more by tumor biology and peri-transplant management, including desensitization intensity and immunosuppression exposure, than by ABO incompatibility itself. **Conclusions:** Current limited comparative evidence does not demonstrate inferior RFS or OS after ABOi LDLT in carefully selected patients with HCC. Larger multicenter comparative studies with standardized reporting of tumor biology, desensitization protocols, and immunosuppression exposure are warranted to confirm these findings and clarify protocol-related effects on post-transplant recurrence.

## Simple Summary

ABO-incompatible living-donor liver transplantation (LDLT) is increasingly used when an ABO-compatible donor is unavailable, but its oncologic safety in hepatocellular carcinoma (HCC) remains debated. In this systematic review, eight studies were included and three comparative studies were quantitatively synthesized. Pooled time-to-event analysis showed no significant difference in recurrence-free survival or overall survival between ABO-incompatible and ABO-compatible LDLT, supporting ABO-incompatible LDLT as an acceptable option in selected patients with HCC.

## 1. Introduction

Hepatocellular carcinoma (HCC) remains one of the leading causes of cancer-related mortality worldwide, and liver transplantation offers the unique advantage of treating both the tumor and the underlying cirrhotic liver [1]. In East Asia, where living-donor liver transplantation (LDLT) is widely practiced due to organ shortage, ABO-incompatible (ABOi) LDLT has emerged as an important alternative when an ABO-compatible (ABOc) donor is unavailable [2,3,4].

With the introduction of rituximab-based desensitization protocols, the safety of ABOi LDLT has improved substantially, leading to its broader clinical adoption. However, a critical concern remains regarding its oncologic safety. ABOi LDLT requires intensified immunologic manipulation, including B-cell depletion and plasma exchange, which may theoretically impair tumor immune surveillance and increase the risk of post-transplant recurrence. This concern is particularly relevant in HCC, where tumor biology and immune interactions play a central role in disease progression [5].

Existing studies evaluating oncologic outcomes after ABOi LDLT are limited by retrospective design, small sample sizes, and substantial overlap of patient cohorts across a few high-volume centers, predominantly in Korea [6,7,8]. Moreover, the available evidence is inconsistent, with some studies suggesting comparable outcomes [6,7,8], while others raise concerns regarding recurrence risk related to peri-transplant immunosuppression and desensitization intensity [9,10,11,12,13,14]. Importantly, no consensus has been reached, and the lack of quantitative synthesis using time-to-event outcomes further limits clinical interpretation.

Given the increasing use of ABOi LDLT and the absence of definitive evidence regarding its oncologic safety, a systematic evaluation of the available data is warranted. Therefore, the present study aimed to perform a systematic review and meta-analysis of comparative studies to determine whether ABO incompatibility is associated with differences in recurrence-free survival (RFS) and overall survival (OS) after LDLT for HCC.

## 2. Methods

### 2.1. Study Design and Protocol

This systematic review and meta-analysis was conducted in accordance with the Preferred Reporting Items for Systematic Reviews and Meta-Analyses (PRISMA) 2020 statement [15]. The completed PRISMA checklist is provided in Supplementary Table S1. The protocol was registered in PROSPERO (registration number: CRD420261367580).

### 2.2. Eligibility Criteria

Studies were eligible if they (i) included adult patients with HCC undergoing LDLT; (ii) compared ABOi and ABOc LDLT for quantitative synthesis, or evaluated HCC-specific oncologic outcomes after ABOi LDLT for qualitative synthesis; and (iii) reported at least one oncologic endpoint, including RFS, OS, tumor recurrence, or time to recurrence.

Case reports, conference abstracts, pediatric-only studies, non-HCC studies, and studies without extractable oncologic data were excluded from quantitative synthesis. Where potentially overlapping institutional cohorts were identified, the most directly relevant HCC-specific comparative study was retained for meta-analysis, and related reports were reserved for qualitative discussion only.

Two reviewers independently screened titles, abstracts, and full texts for eligibility. Disagreements were resolved by discussion, with involvement of a third reviewer when necessary.

### 2.3. Search Strategy and Study Selection

A structured search of PubMed, Embase, and Web of Science was performed using combinations of the following terms: “ABO incompatible”, “ABO-incompatible”, “liver transplantation”, “living donor liver transplantation”, “LDLT”, “hepatocellular carcinoma”, and “HCC”. The full search strategies for all databases are presented in Supplementary Table S2. Reference lists of relevant studies were also reviewed. Study selection was performed in two stages: title/abstract screening followed by full-text review.

### 2.4. Data Extraction

Data extracted included study design, institution, study period, cohort size, matching strategy, recipient and tumor characteristics, follow-up duration, and oncologic outcomes. For quantitative synthesis, hazard ratios (HRs) and 95% confidence intervals (CIs) for RFS and OS were extracted directly when reported. If HRs were not explicitly provided, they were estimated from Kaplan–Meier curves and accompanying log-rank statistics using the method described by Tierney et al. [16]. Two reviewers independently extracted data using a predefined data collection form, and discrepancies were resolved by consensus.

### 2.5. Risk-of-Bias Assessment

The methodological quality of comparative cohort studies was assessed using the Newcastle–Ottawa Scale [17]. Because all included comparative studies were retrospective single-center cohorts, selection bias and residual confounding were considered inherent risks.

### 2.6. Statistical Analysis

Random-effects meta-analysis was performed for RFS and OS using pooled HRs and 95% CIs. Between-study heterogeneity was assessed using the I^2^ statistic. Given the limited number of comparative studies, no formal publication-bias analysis was performed. All analyses were conducted in R (version 4.3.2; R Foundation for Statistical Computing, Vienna, Austria) using the meta and metafor packages. Forest plots and summary figures were generated in R, and the PRISMA 2020 flow diagram was created using Microsoft PowerPoint 365.

## 3. Results

### 3.1. Study Selection

**Figure 1:**
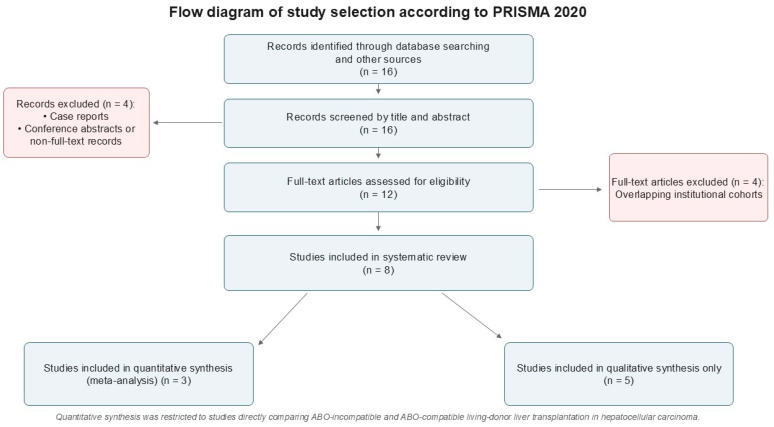
PRISMA 2020 flow diagram summarizing study identification, screening, eligibility assessment, and final inclusion.

A total of 16 reports were identified through database searching and citation tracking. After screening, 12 full-text articles were assessed for eligibility. Four reports were excluded at the full-text stage because of overlapping institutional cohorts. Ultimately, 8 studies were included in the systematic review, of which 3 comparative studies were eligible for quantitative synthesis and 5 additional studies were included in qualitative synthesis (Figure 1). Detailed screening results and reasons for exclusion are provided in Supplementary Table S3. Risk-of-bias assessment, cohort adjudication for overlapping populations, and detailed data extraction for the meta-analysis are presented in Supplementary Tables S4–S6.

### 3.2. Characteristics of Included Comparative Studies

The three comparative studies originated from Asan Medical Center, Samsung Medical Center, and the National Cancer Center, Korea (Table 1) [6,7,8]. All were retrospective single-center cohort studies. The Asan study by Yoon et al. included the largest matched cohort (165 ABOi vs. 165 ABOc) [6], whereas Kim JM et al. and Kim SH et al. included 59 ABOi versus 181 ABOc recipients and 39 ABOi versus 78 ABOc recipients, respectively [7,8]. Two studies used propensity score matching, and one reported both unmatched and matched analyses. The included studies were conducted in high-volume centers in Korea, reflecting the regional predominance of ABOi LDLT. Across studies, baseline tumor characteristics and recipient profiles were generally well balanced between ABOi and ABOc groups, particularly in propensity-matched analyses. Follow-up duration varied across studies, ranging from approximately 28 to 48 months, including both mean and median estimates.

### 3.3. Recurrence-Free Survival

**Figure 2:**
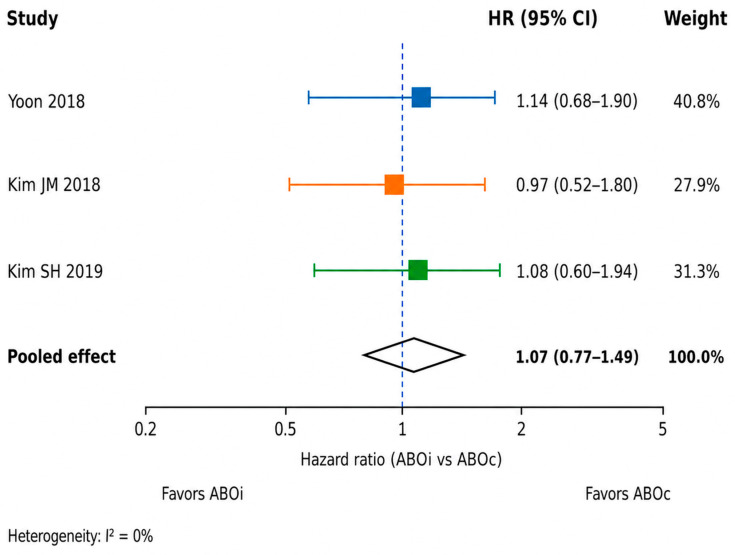
Forest plot of recurrence-free survival comparing ABO-incompatible versus ABO-compatible living-donor liver transplantation for hepatocellular carcinoma [6,7,8]. Abbreviations: ABOi, ABO-incompatible; ABOc, ABO-compatible; HR, hazard ratio; CI, confidence interval.

Meta-analysis of three comparative studies demonstrated no significant difference in RFS between ABOi and ABOc LDLT (HR 1.07, 95% CI 0.77–1.49; I^2^ = 0%) (Figure 2). All study-specific estimates were centered around unity, with no evidence of between-study heterogeneity.

### 3.4. Overall Survival

**Figure 3:**
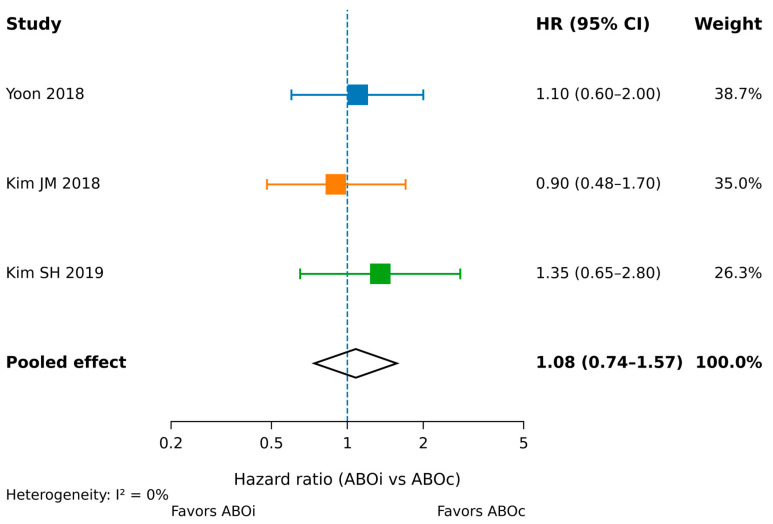
Forest plot of overall survival comparing ABO-incompatible versus ABO-compatible living-donor liver transplantation for hepatocellular carcinoma [6,7,8]. Abbreviations: ABOi, ABO-incompatible; ABOc, ABO-compatible; HR, hazard ratio; CI, confidence interval.

Pooled analysis similarly showed no significant difference in OS between ABOi and ABOc LDLT (HR 1.08, 95% CI 0.74–1.57; I^2^ = 0%) (Figure 3). Between-study heterogeneity was negligible.

### 3.5. Sensitivity Analysis

Leave-one-out sensitivity analyses demonstrated that exclusion of any single study did not materially alter the pooled estimates for either RFS or OS (Supplementary Figures S1 and S2).

### 3.6. Qualitative Synthesis

Five non-comparative or exploratory studies were synthesized qualitatively (Table 2). Early historical reports suggested technical feasibility but were limited by small sample sizes [9,18]. More recent studies provided insights into potential determinants of recurrence, highlighting the role of peri-transplant factors such as the intensity of desensitization protocols, including plasma exchange, and the level of early post-transplant immunosuppression, particularly tacrolimus exposure [10,11,12]. Collectively, these studies consistently suggested that recurrence risk may be more strongly influenced by tumor biology and peri-transplant management rather than ABO incompatibility itself, supporting the findings of the quantitative synthesis. An exploratory event-based meta-analysis of crude recurrence risk yielded consistent findings, although it was not included in the primary analysis due to the lack of time-to-event data (Supplementary Figure S3).

## 4. Discussion

This systematic review and meta-analysis synthesizes the limited comparative evidence on oncologic outcomes after ABOi versus ABOc LDLT for HCC. Given the fragmented nature of the existing literature, it represents one of the first quantitative syntheses focused specifically on HCC outcomes after ABOi LDLT. Across three comparative cohorts from high-volume Korean centers, ABO incompatibility was not associated with worse RFS or OS, and statistical heterogeneity was negligible [6,7,8]. These findings were further supported by leave-one-out sensitivity analyses, in which omission of any single study did not materially alter the pooled estimates (Supplementary Figures S1 and S2). Taken together, the available comparative evidence does not support the concern that ABO incompatibility per se leads to inferior long-term oncologic outcomes after LDLT for HCC.

These findings are clinically important because ABOi LDLT is not used in an abstract setting, but rather in situations where access to timely transplantation is limited by donor availability [2]. In regions where deceased-donor grafts remain scarce and LDLT is the dominant transplant strategy, the ability to safely use an ABOi donor may shorten time to transplantation, reduce the risk of tumor progression while waiting, and expand access to potentially curative therapy [3,4]. From this perspective, the present results suggest that ABO incompatibility itself should not be regarded as an automatic oncologic contraindication in appropriately selected patients with HCC [6,7,8].

The present study also addresses a long-standing biologic concern: whether rituximab-based desensitization, plasma exchange, and intensified peri-transplant immunosuppression might impair anti-tumor immune surveillance and thereby increase post-transplant recurrence. Tumor immune interactions have been reported to play a central role in HCC progression [5]. Although this concern is biologically plausible, the available comparative data do not demonstrate a clinically meaningful adverse effect of ABO incompatibility on RFS or OS [6,7,8]. Instead, the comparative studies suggest that established tumor-related factors remain the dominant determinants of recurrence, including tumor burden, alpha-fetoprotein level, encapsulation, and microvascular invasion, rather than ABO status itself. These findings are consistent with a previous study demonstrating that tumor biology plays a central role, while host inflammatory and immunological conditions may also influence post-transplant recurrence [13].

The qualitative studies included in this review provide further context for this interpretation. Early reports primarily established the technical feasibility of ABOi LDLT in HCC [18], but more recent non-comparative studies suggest that recurrence risk may be influenced more by peri-transplant management intensity than by ABO incompatibility itself. In particular, recent cohorts have highlighted the potential importance of factors such as the extent of plasma exchange [12] and early tacrolimus exposure [11]. Consistent with this, the association between calcineurin inhibitor exposure and an increased risk of HCC recurrence has also been demonstrated in a previous study [14], suggesting that protocol-level variables may be more relevant modulators of recurrence than the ABO barrier alone. Thus, the clinically relevant question may be less whether ABOi LDLT is intrinsically oncologically unsafe, and more how desensitization and immunosuppression should be optimized in biologically high-risk HCC.

The supplementary analyses strengthen this conclusion. The exploratory event-based meta-analysis of crude recurrence events (Supplementary Figure S3) did not materially alter the overall interpretation that a clear increase in recurrence risk was not demonstrated. However, that analysis was appropriately treated as supplementary because crude risk ratios do not account for censoring or variable follow-up and therefore are methodologically less appropriate than time-to-event effect estimates for the primary synthesis. Likewise, the rationale for excluding potentially overlapping institutional cohorts from the pooled analysis is transparently detailed in Supplementary Table S5, and the study-level extraction decisions used for quantitative synthesis are summarized in Supplementary Table S6. In a literature of this size, such adjudication is essential to avoid double counting and to preserve the interpretability of pooled estimates.

From a practical standpoint, these findings support the selective use of ABOi LDLT in patients with HCC when timely transplantation is critical [2,3,4]. This is particularly relevant in transplant environments where ABOc living donors are unavailable and delay may result in tumor progression or loss of transplant eligibility. The current evidence suggests that, when modern desensitization protocols are used and appropriate oncologic selection is maintained, expansion of the donor pool through ABOi LDLT may be achieved without clear evidence of inferior oncologic outcomes [6,7,8]. Accordingly, ABOi LDLT should be considered primarily in carefully selected HCC candidates, such as patients within accepted transplant criteria, with controlled tumor burden and favorable tumor biology, and without an available ABO-compatible living donor. These procedures should preferably be performed in experienced high-volume centers with standardized desensitization protocols, careful immunosuppression monitoring, and multidisciplinary assessment of recurrence risk.

The present findings should also be interpreted within the broader multidisciplinary management of HCC. Locoregional therapies, particularly transarterial chemoembolization (TACE), are frequently used for disease control, bridging, or downstaging before definitive treatment, while liver resection remains an important curative option in selected patients [19]. In rare cases of large HCC, paraneoplastic manifestations such as hypoglycemia may improve after tumor-directed therapies, including chemoembolization or resection [20]. The management of HCC with portal vein tumor thrombosis remains controversial; although it is generally considered an adverse oncologic feature, surgical treatment may be reconsidered in carefully selected patients within multidisciplinary strategies [21]. Beyond conventional clinical factors, emerging tools such as artificial intelligence and image processing may improve diagnosis, treatment selection, and prognostic modeling in liver cancer, particularly by integrating radiologic tumor phenotypes with biologic risk factors [22]. Such approaches may help refine recurrence-risk stratification in HCC candidates undergoing LDLT, although they have not yet been specifically validated for ABOi LDLT. In addition, experimental and translational studies suggest that innate immune pathways, including Toll-like receptor signaling, may contribute to liver regeneration after hepatectomy, although these mechanisms have not been directly evaluated in ABOi LDLT for HCC [23]. Experience from liver-directed therapies for secondary liver malignancies, including colorectal liver metastasis, also highlights the importance of balancing oncologic control with treatment-related morbidity, including rare fistulous complications after radiation-based approaches [24].

Several limitations should be acknowledged. First, the certainty of the available evidence should be considered low because the meta-analysis included only three retrospective single-center comparative studies, all from Korea. Although propensity score matching was used in two studies, residual confounding remains possible. ABOi and ABOc recipients may have differed in donor availability, waiting time, tumor burden, alpha-fetoprotein level, tumor biology, and transplant urgency. These factors may have either masked or exaggerated the true association between ABO incompatibility and recurrence risk. Second, ABOi LDLT is not a uniform exposure. Outcomes may be influenced by center-specific desensitization and immunosuppression protocols, including rituximab use, plasma exchange intensity, and early post-transplant tacrolimus exposure. These protocol-level factors may confound or modify the relationship between ABO incompatibility and post-transplant recurrence. Because the included studies did not provide sufficiently granular patient-level data, the present analysis could not separate the independent effect of ABO incompatibility from peri-transplant management factors. Third, two pooled effect estimates required derivation from Kaplan–Meier curves rather than direct reporting of hazard ratios, which is methodologically acceptable but less robust than published multivariable time-to-event estimates. Finally, because of the small number of observational studies, formal publication-bias assessment and formal GRADE evaluation were not performed. These limitations, together with the study-level risk-of-bias profile summarized in Supplementary Table S4, indicate that the present findings should be interpreted as supportive rather than definitive.

Despite these limitations, this review has several notable strengths. It focuses specifically on HCC rather than mixed LDLT populations, prioritizes time-to-event oncologic outcomes over crude event counts, systematically adjudicates overlapping cohorts, and integrates both quantitative and qualitative evidence into a clinically coherent framework. Importantly, the convergence of the primary meta-analysis, the leave-one-out sensitivity analyses, and the supplementary exploratory recurrence analysis supports the overall conclusion that ABO incompatibility itself has not been shown to worsen post-transplant oncologic outcomes in HCC.

Future studies should move beyond small retrospective single-center cohorts and aim for multicenter collaborative datasets with standardized reporting of desensitization protocols, tumor biology, and time-to-event outcomes. Such efforts would help clarify whether specific subgroups, including patients beyond the Milan criteria or those requiring more intensive desensitization, have differential oncologic risk. Until such data become available, the current evidence supports the view that, in carefully selected patients, ABOi LDLT can expand access to potentially curative transplantation without clear evidence of inferior RFS or OS.

## 5. Conclusions

Current limited comparative evidence does not demonstrate inferior RFS or OS after ABOi LDLT in selected patients with HCC. However, because the available data are derived from a small number of retrospective single-center cohorts, these findings should not be interpreted as definitive oncologic equivalence. In experienced centers using standardized desensitization and peri-transplant management, ABOi LDLT may be considered for carefully selected HCC candidates when an ABOc donor is unavailable. Larger multicenter studies with standardized reporting of tumor biology, desensitization intensity, immunosuppression exposure, and time-to-event outcomes are warranted to confirm these findings and clarify protocol-related effects on post-transplant recurrence.

## Figures and Tables

**Table 1:** Characteristics of studies included in the quantitative synthesis.

Study	Center	Design	Cohort	Matching	Primary Oncologic Data	Key Finding
Yoon et al., 2018 [6]	Asan Medical Center	Retrospective cohort	165 ABOi vs. 165 ABOc	1:1 propensity score-matched cohort reported	RFS HR 1.14 (95% CI 0.68–1.90); OS HR 1.10 (95% CI 0.60–2.00)	Comparable RFS and OS
Kim JM et al., 2018 [7]	Samsung Medical Center	Retrospective cohort	59 ABOi vs. 181 ABOc	Unmatched comparative cohort	1-, 2-, and 3-year DFS/OS; HR estimated from KM curves	Comparable recurrence and survival
Kim SH et al., 2019 [8]	National Cancer Center Korea	Retrospective cohort	39 ABOi vs. 78 ABOc	1:2 propensity score-matched cohort	1-, 3-, and 5-year RFS/OS; HR estimated from KM curves	Comparable RFS and OS

Abbreviations: ABOi, ABO-incompatible; ABOc, ABO-compatible; DFS, disease-free survival; HR, hazard ratio; KM, Kaplan–Meier; OS, overall survival; RFS, recurrence-free survival.

**Table 2:** Studies included in qualitative synthesis only.

Study	Center/Era	Design	Reason for Qualitative-Only Inclusion	Main Message
Matsuno et al., 2008 [18]	Japan	Case series	No comparator; small historical cohort	Early feasibility of ABOi LDLT for HCC
Miyagi et al., 2012 [9]	Japan	Exploratory cohort	No direct ABOi vs. ABOc comparative effect estimate	Recurrence may be linked to immunosuppression intensity
Oh et al., 2023 [10]	Samsung Medical Center	ABOi HCC cohort	Potential cohort overlap with Samsung comparative study; no ABOc comparator	Post-transplant plasma exchange associated with improved RFS in selected subgroups
Han et al., 2023 [11]	Catholic Medical Center	ABOi HCC cohort	No ABOc comparator	Higher early tacrolimus exposure associated with recurrence
Yoo et al., 2025 [12]	Yonsei University	ABOi HCC cohort	No ABOc comparator	Greater number of pre-transplant plasma exchange sessions associated with recurrence risk

Abbreviations: ABOi, ABO-incompatible; ABOc, ABO-compatible; LDLT, living donor liver transplantation; HCC, hepatocellular carcinoma.

## Data Availability

The data analyzed in this study were derived from published articles. Additional working extraction files are available from the corresponding author upon reasonable request.

## Supplementary Materials

The following supporting information can be downloaded at: https://www.mdpi.com/article/10.3390/cancers18111687/s1, Figure S1: Leave-one-out sensitivity analysis for recurrence-free survival; Figure S2: Leave-one-out sensitivity analysis for overall survival; Figure S3: Exploratory event-based meta-analysis of crude recurrence risk; Table S1: PRISMA 2020 checklist summary for the present review; Table S2: Full electronic search strategies; Table S3: Full-text articles excluded after eligibility assessment, with reasons; Table S4: Risk-of-bias assessment using the Newcastle–Ottawa Scale (cohort studies); Table S5: Overlapping cohort adjudication and final study assignment; Table S6: Quantitative data extraction sheet for the primary meta-analysis.

## Abbreviations

ABOi, ABO-incompatible; ABOc, ABO-compatible; LDLT, living donor liver transplantation; HCC, hepatocellular carcinoma; RFS, recurrence-free survival; OS, overall survival; HR, hazard ratio; CI, confidence interval; PRISMA, Preferred Reporting Items for Systematic Reviews and Meta-Analyses.
