# Supplementary material for: Oncologic Outcomes After ABO-Incompatible Versus Compatible Living Donor Liver Transplantation for Hepatocellular Carcinoma: A Systematic Review and Meta-Analysis
Source: Cancers (Basel). 2026 May 22;18(11):1687. doi: 10.3390/cancers18111687 (PMC13255586; doi:10.3390/cancers18111687)
Supplement: Supplementary file 1 [file cancers-18-01687-s001.zip › cancers-4289054-supplementary.pdf]

Supplementary Material: Oncologic Outcomes After ABO-Incompatible Versus Compatible Living Donor Liver Transplantation for Hepatocellular Carcinoma: A Systematic Review and Meta-Analysis

Seoung Hoon Kim, Byeong Ho An, Jin A Lee and Go Woon Jeong

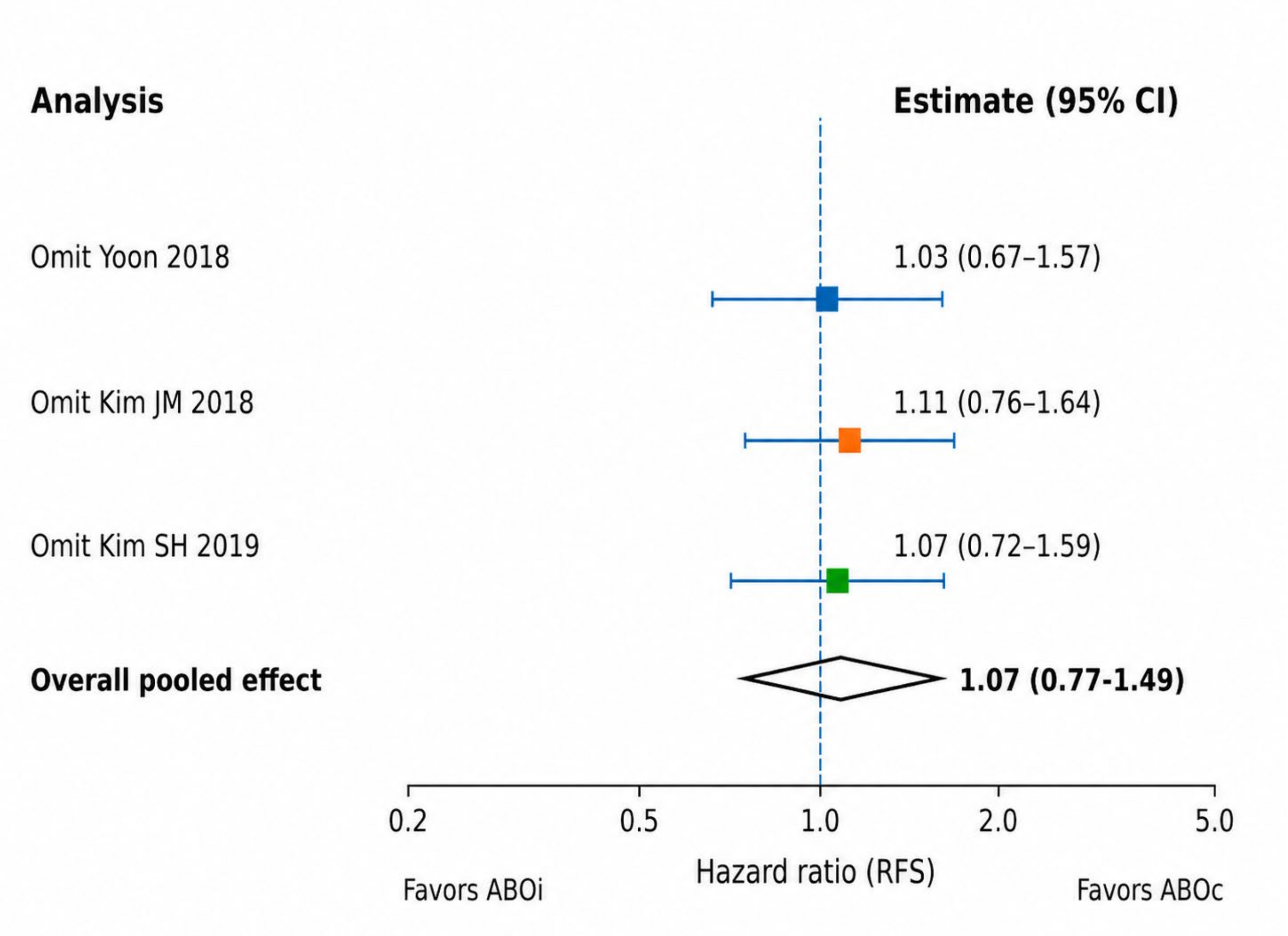

Figure S1. Leave-one-out sensitivity analysis for recurrence-free survival. Pooled hazard ratio for recurrence-free survival recalculated after sequential omission of each included comparative study. Directional consistency across the omitted-study analyses supports robustness of the primary recurrence-free survival result. Abbreviations**:** ABOi, ABO-incompatible; ABOc, ABO-compatible; RFS, recurrence-free survival; CI, confidence interval.

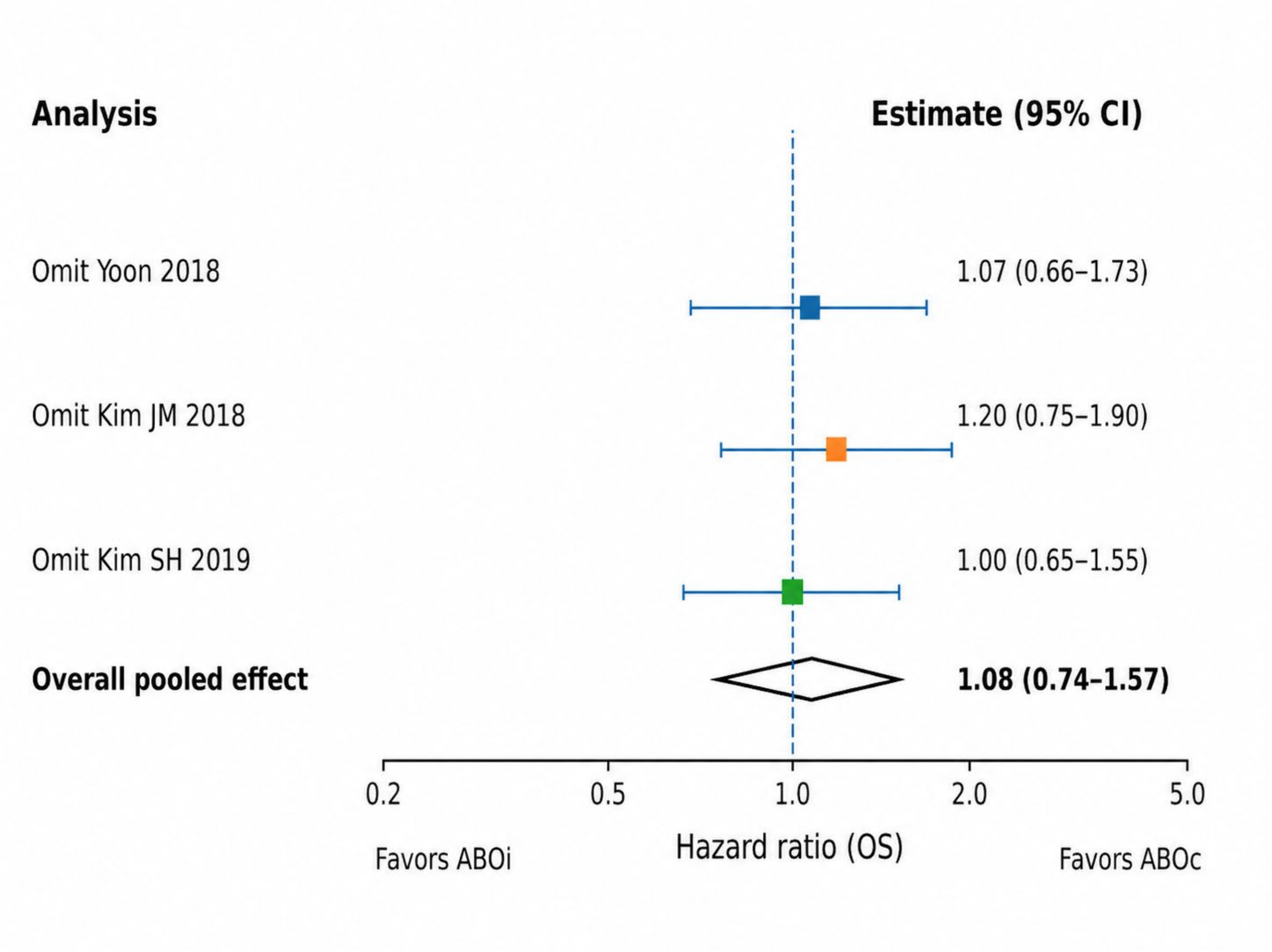

Figure S2. Leave-one-out sensitivity analysis for overall survival. Pooled hazard ratio for overall survival recalculated after sequential omission of each included comparative study. No single study materially altered the direction or significance of the pooled estimate. Abbreviations**:** ABOi, ABO-incompatible; ABOc, ABO-compatible; OS, overall survival; CI, confidence interval.

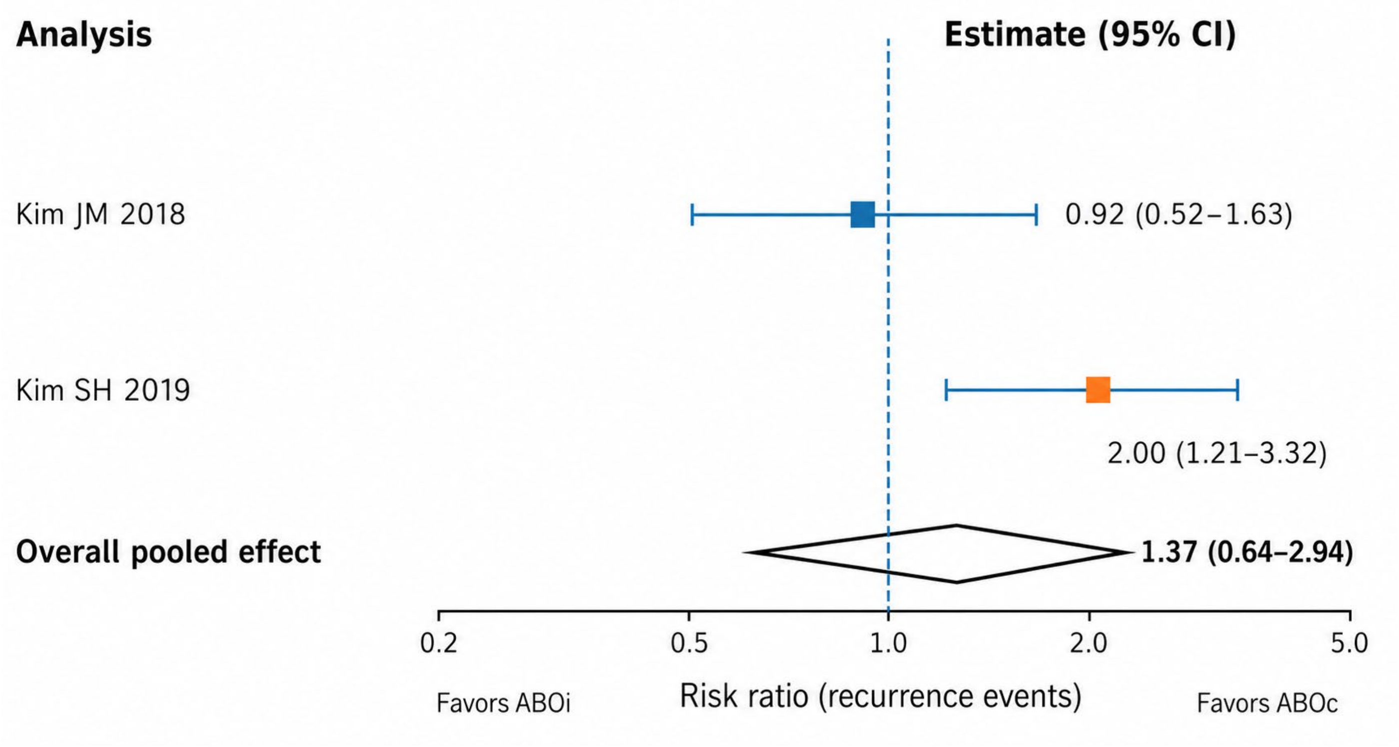

Figure S3. Exploratory event-based meta-analysis of crude recurrence risk. Exploratory random-effects meta-analysis of crude recurrence events using risk ratios. This analysis was considered supplementary because event-based risk ratios do not account for censoring or variable follow-up and were therefore not used as the primary synthesis metric. Abbreviations: ABOi, ABO-incompatible; ABOc, ABO-compatible; CI, confidence interval.

Table S1. PRISMA 2020 checklist summary for the present review.

Section	Item	Checklist item	Location in manuscript
Title	1	Identify the report as a systematic review and meta-analysis	Title page
Abstract	2	Provide a structured summary including background, methods, results, and conclusions	Abstract
Introduction	3	Describe the rationale for the review in the context of existing knowledge	Introduction
Introduction	4	Provide an explicit statement of the objectives or research questions	Introduction
Methods	5	Specify inclusion and exclusion criteria	Methods (Eligibility criteria)
Methods	6	Specify all information sources and date of last search	Methods (Search strategy)
Methods	7	Present full search strategies for all databases	Supplementary Table S2
Methods	8	Describe the selection process (screening, eligibility)	Methods (Study selection)
Methods	9	Describe data collection process	Methods (Data extraction)
Methods	10	List and define all outcomes	Methods (Data extraction; outcomes defined)
Methods	11	Describe methods for assessing risk of bias	Methods (Risk of bias assessment)
Methods	12	Specify effect measures (e.g., HR, CI)	Methods (Statistical analysis)
Methods	13	Describe synthesis methods, including model and heterogeneity assessment	Methods (Statistical analysis)
Methods	14	Describe methods to assess reporting bias	Not performed due to limited number of studies
Methods	15	Describe methods to assess certainty of evidence	Not performed

Results	16	Describe results of study selection process	Results; Figure 1
Results	17	Cite included studies and present characteristics	Results; Tables 1 and 2
Results	18	Present risk of bias assessment results	Supplementary Table S4
Results	19	Present results of individual studies	Results (Recurrence-Free Survival; Overall Survival)
Results	20	Present results of syntheses (meta-analysis)	Results; Figures 2 and 3
Discussion	21	Provide general interpretation of the results	Discussion
Discussion	22	Discuss limitations of the evidence and review process	Discussion
Other information	23	Provide registration information	Methods (PROSPERO registration: CRD420261367580)
Other information	24	Describe sources of support	Funding statement
Other information	25	Declare competing interests	Conflicts of Interest
Other information	26	Provide data availability statement	Data Availability Statement

Table S2. Full electronic search strategies.

Database	Search date	Search strategy
PubMed	2026-04-13	("ABO incompatible"[Title/Abstract] OR "ABO-incompatible"[Title/Abstract] OR "ABO incompatibility"[Title/Abstract]) AND ("living donor liver transplantation"[Title/Abstract] OR LDLT[Title/Abstract] OR "liver transplantation"[Title/Abstract]) AND ("hepatocellular carcinoma"[Title/Abstract] OR HCC[Title/Abstract])
Embase	2026-04-13	('abo incompatibility':ti,ab OR 'abo incompatible':ti,ab OR 'abo-incompatible':ti,ab) AND ('living donor liver transplantation':ti,ab OR ldlt:ti,ab OR 'liver transplantation':ti,ab) AND ('hepatocellular carcinoma':ti,ab OR hcc:ti,ab)
Web of Science	2026-04-13	TS=("ABO incompatible" OR "ABO-incompatible" OR "ABO incompatibility") AND TS=("living donor liver transplantation" OR LDLT OR "liver transplantation") AND TS=("hepatocellular carcinoma" OR HCC)

Table S3. Full-text articles excluded after eligibility assessment, with reasons.

Study	Institution/program	Reason for exclusion from quantitative synthesis
Kim JM et al., 2013	Samsung Medical Center	Potentially overlapping institutional program; replaced by the more directly relevant HCC comparative study by Kim JM et al., 2018.
Lee SD et al., 2014	National Cancer Center Korea	Potential overlap with the later HCC-specific

Study	Institution/program	Reason for exclusion from quantitative synthesis
		comparative cohort; retained only for overlap adjudication.
Lee SD et al., 2015	National Cancer Center Korea	General/immunologic ABO-incompatible LDLT cohort with likely overlap; not HCC-comparative for meta-analysis.
Song GW et al., 2014	Asan Medical Center	General adult ABO-incompatible LDLT cohort with likely institutional overlap; replaced by the HCC-specific comparative study by Yoon YI et al., 2018.

Abbreviations**:** HCC, hepatocellular carcinoma; LDLT, living donor liver transplantation.

Table S4. Risk-of-bias assessment using the Newcastle–Ottawa Scale (cohort studies).

Study	Selection (max 4)	Comparability (max 2)	Outcome (max 3)	Total
Yoon YI et al., 2018	4	2	3	9
Kim JM et al., 2018	4	1	3	8
Kim SH et al., 2019	4	2	2	8

Table S5. Overlapping cohort adjudication and final study assignment.

Institution	Candidate studies	Potential overlap	Final assignment
Samsung Medical Center	Kim JM 2013; Kim JM 2018; Oh 2023	Yes	Kim JM 2018 retained for quantitative synthesis; Oh 2023 retained for qualitative synthesis only; Kim JM 2013 excluded.
National Cancer Center Korea	Lee SD 2014; Lee SD 2015; Kim SH 2019	Yes	Kim SH 2019 retained for quantitative synthesis; earlier cohorts excluded from meta-analysis.

Institution	Candidate studies	Potential overlap	Final assignment
Asan Medical Center	Song GW 2014; Yoon YI 2018	Yes	Yoon YI 2018 retained for quantitative synthesis; Song GW 2014 excluded from meta-analysis.
Catholic Medical Center	Han JW 2023	No evident overlap with main comparative set	Qualitative synthesis only (no ABO-compatible comparator).
Yonsei University	Yoo YJ 2025	No evident overlap with main comparative set	Qualitative synthesis only (no ABO-compatible comparator).

Table S6. Quantitative data extraction sheet for the primary meta-analysis.

Study	ABOi n	ABOc n	Matching	RFS effect estimate	OS effect estimate	Source of estimate
Yoon YI et al., 2018	165	165	1:1 propensity matched	HR 1.14 (95% CI 0.68–1.90)	HR 1.10 (95% CI 0.60–2.00)	Directly reported in article/abstract
Kim JM et al., 2018	59	181	No propensity matching	KM-derived HR used for primary synthesis	KM-derived HR used for primary synthesis	Reconstructed from published Kaplan–Meier and log-rank information
Kim SH et al., 2019	39	78	1:2 propensity matched	KM-derived HR used for primary synthesis	KM-derived HR used for primary synthesis	Reconstructed from published Kaplan–Meier and log-rank information

Abbreviations: ABOi, ABO-incompatible; ABOc, ABO-compatible; RFS, recurrence-free survival; OS, overall survival; HR, hazard ratio; CI, confidence interval; KM, Kaplan–Meier.
